# The Optimal Management of Fistulizing Crohn’s Disease: Evidence beyond Randomized Clinical Trials

**DOI:** 10.3390/jcm11113045

**Published:** 2022-05-28

**Authors:** Panu Wetwittayakhlang, Alex Al Khoury, Gustavo Drügg Hahn, Peter Laszlo Lakatos

**Affiliations:** 1 Division of Gastroenterology, McGill University Health Center, Montreal, QC H3G 1A4, Canada or wet.panu@gmail.com (P.W.); gustavodhahn@gmail.com (G.D.H.); 2 Gastroenterology and Hepatology Unit, Division of Internal Medicine, Faculty of Medicine, Prince of Songkla University, Songkhla 90110, Thailand; 3 Division of Gastroenterology, University of Florida, Jacksonville, FL 32209, USA; alexkhoury88@gmail.com; 4 Graduate Course Sciences in Gastroenterology and Hepatology, School of Medicine, Universidade Federal do Rio Grande do Sul, Porto Alegre 90035-002, Brazil; 5 First Department of Medicine, Semmelweis University, 1085 Budapest, Hungary

**Keywords:** fistulizing, Crohn’s disease, perianal fistula, internal fistula, anti-TNF, biologic, stem cells therapy

## Abstract

Fistulizing Crohn’s disease (FCD) remains the most challenging aspect of treating patients with CD. FCD can occur in up to 30% of patients with CD and may lead to significant disability and impaired quality of life. The optimal treatment strategies for FCD require a multidisciplinary approach, including a combined medical and surgical approach. The therapeutic options for FCD are limited due to sparse evidence from randomized clinical trials (RCTs). The current recommendations are mainly based on post hoc analysis from RCTs, real-world clinical studies and expert opinion. There is variation in everyday clinical practice amongst gastroenterologists and surgeons. The evidence for anti-tumor necrosis factor therapy is the strongest in the treatment of FCD. However, long-term fistula healing can be achieved in only 30–50% of patients. In recent years, emerging data in the advent of therapeutic modalities, including the use of new biologic agents, therapeutic drug monitoring, novel surgical methods and mesenchymal stem cell therapy, have been shown to improve outcomes in achieving fistula healing. This review summarizes the existing literature on current and emerging therapies to provide guidance beyond RCTs in managing FCD.

## 1. Introduction

A fistula is a common complication and aggressive phenotype of Crohn’s disease (CD) that affects approximately 30% of patients during their disease course [1,2,3]. Fistulas in CD can be classified into internal and external fistulas. Internal fistulas connect the bowel and the internal organs and include enteroenteric, enterocolonic, enterovaginal and enterovesical fistulas. External fistulas that connect to the skin include perianal and enterocutaneous fistulas. Fistulizing Crohn’s disease (FCD) comprises perianal (55%), entero-enteric (24%), rectovaginal (9%), enterocutaneous (6%), enterovesical (3%) and entero-intraabdominal (3%) fistulas [4]. The perianal fistula is the most common type, and approximately 20% of patients with CD develop perianal disease within 10 years of a CD diagnosis, including 11% who have perianal disease at presentation [5]. Perianal disease activity often correlates with luminal CD, although 5% of patients will have isolated perianal disease without luminal inflammation [6].

FCD is associated with significant disability as well as physical and psychological impairments to quality of life [7,8,9]. The optimal treatment strategies for FCD require a multidisciplinary approach and remain challenging. However, medical and surgical management for FCD differs in everyday clinical practice. A national survey of gastroenterologists and surgeons in the UK with respect to managing perianal fistulas demonstrated a huge variation in clinical practice, including the medical therapies deployed and choices of surgical management [10,11]. Moreover, FCD is difficult to treat and frequently recurs. The rates of failed fistula healing with medical treatments were reported up to be 70%, and 40% of patients had relapses in the long term [12].

Randomized clinical trials (RCTs) with a primary endpoint of treating FCD are scarce. Therefore, the current evidence is based mainly on the post-hoc analysis of RCTs, retrospective or observational studies and expert opinion [13]. Furthermore, given the limited comparative studies, the current practice guidelines have provided only weak recommendations in terms of therapeutic options [14,15,16,17,18].

This review aims to provide updated evidence based on RCTs and highlights data beyond RCTs in the realm of therapeutic options, including strategies to optimize responses to anti-TNF therapy, a combination of anti-TNF with immunomodulators and/or biologics, new biological/small molecules therapy, surgical treatment and mesenchymal stem cell therapy in treating FCD. We discuss existing evidence on the efficacy and treatment outcomes of each therapeutic option, strategies to optimize fistula healing and propose an algorithm for managing patients with FCD.

## 2. Medical Treatment for Fistulizing CD

Current management strategies for FCD aim to induce fistula healing, control complications (infection and sepsis) and improve quality of life. The current clinical guidelines recommend a multidisciplinary approach, including medical treatment combined with seton or surgical drainage [14,15,16,17,18,19].

In the context of FCD, infliximab (IFX) is the only biologic that has proven efficacy in randomized placebo-controlled trials as a specific indication for its use in treating FCD. Nevertheless, other biologics, including adalimumab (ADA), ustekinumab (UST) and vedolizumab (VDZ) were noted to have efficacy, supporting their use in the current practice guideline [15].

In contrast, there is no role for corticosteroids or aminosalicylates in the treatment of FCD. Steroids are ineffective in fistula healing and may result in increased sepsis, a worsening of discharge and an increased need for surgery. Steroids may be used to control active luminal disease, but adequate drainage of a perianal abscess is very important for source control and preventing infection [18]. In addition, cyclosporine and mycophenolate mofetil should not be used for treating FCD. Tacrolimus may benefit in treating FCD in the short-term, but fistula closure rates reported in RCTs were very low, and significant toxicity precludes long-term use with this agent [16,19,20].

### 2.1. What Is the Evidence of Anti-TNF Therapy from RCTs and beyond RCT?

Anti-TNF has become the cornerstone of therapy for FCD. Several randomized, placebo-controlled trials have demonstrated the efficacy of anti-TNF therapy for the induction and maintenance of remission in FCD. Thus, current evidence suggests the use of anti-TNF therapy as a first-line treatment for FCD [14]. However, evidence indicates that certolizumab pegol may not be effective in the induction of fistula remission. The selected key studies in FCD are summarized in Table 1.

#### 2.1.1. Infliximab

Infliximab (IFX) has been proven to be the first-line and most efficacious biologic treatment for symptomatic perianal CD according to randomized placebo-controlled trials. There are two RCTs that investigated the efficacy of IFX for FCD [21,22].

In the initial RCT, Present et al. reported 94 patients with FCD. The overall response rate, defined as a ≥ 50% reduction of draining fistulas from the baseline, was 62% with IFX compared to 26% with the placebo (*p* = 0.002). Complete response, defined as an absence of drainage on two consecutive visits, was 46% with IFX vs. 13% with placebo (*p* = 0.001) [21].

In the ACCENT II study, Sands et al. investigated the utility of IFX as a maintenance therapy in 282 patients (87% with perianal CD). The initial response rate to induction therapy was 195/282 (67%) at week 14. The patients that responded were then randomized to receive either IFX or the placebo. For the patients who received maintenance IFX, there was a significantly longer time period before loss of response than for those receiving the placebo (>40 weeks vs. 14 weeks, *p* < 0.001). At 54 weeks, patients with maintenance IFX were more likely to have a complete response (36% vs. 19%; *p* = 0.009). This study suggests that loss of response may occur in approximately half of the patients within 1 year, even amongst those who initially responded to IFX induction and maintenance [22].

In a real-world study, including a retrospective cohort of 178 CD patients with draining perianal fistulas treated with IFX, clinical remission and response rates were observed in 55% and 26% of patients, respectively. On a follow-up radiological response using MRI, 38% of patients had deep radiological remission and 34% had a partial response [25].

#### 2.1.2. Adalimumab

There have been three randomized, placebo-controlled trials investigating the effect of adalimumab (ADA) on fistula healing in the secondary endpoint. Unfortunately, fistula remission has not been a primary endpoint for any RCT involving ADA. In Classic I, Hanauer et al. investigated 299 anti-TNF naïve CD patients. Only 11% had perianal CD at the baseline. There was no difference in fistula healing rates among the groups at week 4. [27] In GAIN, Sandborn et al. reported a placebo-controlled trial in 325 patients who had loss of response from IFX, with 45 patients (11%) having perianal CD. The rates of fistula healing at week 4 were similar for both groups (IFX 20% vs. placebo 15%). [28] However, the results from these studies were evaluated within a short time period and both had a limited number of patients.

The CHARM study, in 2007, was the first study to investigate the efficacy of ADA in the treatment of FCD. Colombel et al. assessed the maintenance therapy in 778 CD patients; 117 (15.2%) patients had draining enterocutaneous or perianal fistulas. Fistula closure was achieved in 30% of the patients treated with ADA, compared to 13% with the placebo, at week 26 (*p* = 0.043) and week 56 (ADA 33% vs. placebo 13%, *p* = 0.016) [29].

In an open-label follow-up observational cohort of the CHARM fistula post-hoc cohort, 90% of patients who had fistula healing at 56 weeks maintained fistula healing a year later, suggesting that complete fistula healing was sustained for up to 2 years by most patients [30]. The CHOICE trial was an open-label, single-arm trial where 673 patients who had failed IFX therapy were enrolled and treated with ADA induction and maintenance therapy, with 88 (13%) patients having enterocutaneous or perianal fistulas. In total, 34/88 (39%) patients achieved complete fistula healing at the time of their last visit (range 4–36 weeks) [32].

In a real-world retrospective study of 46 patients naïve to anti-TNF therapy treated with ADA, fistula response was achieved in 72% and 49% of patients at 6 and 12 months, respectively [33].

In addition, there was no clinical difference observed in terms of efficacy between IFX and ADA for treating FCD in real-world retrospective studies that compared efficacy between both anti-TNF agents [34,48]. In retrospective studies of 47 CD patients with perianal fistulas, there was no significant difference in the rate of being recurrence-free or the aggravation of fistulas (62.5%; ADA vs. 83.9; IFX, *p* = 0.09) at 24 months after treatment [34].

#### 2.1.3. Certolizumab Pegol

Based on negative results from two RCTs, the current evidence to support certolizumab in treating FCD is low compared to other anti-TNF agents. In PRECISE I, a placebo-controlled trial for certolizumab, 107 patients had fistulizing CD; there was no difference in fistula healing at 6 weeks after induction (30% certolizumab vs. 31% placebo) [49]. Thereafter, in PRECISE-2, in 58 patients (15%) with FCD who had an initial response, there was no significant difference in fistula closure between the groups (54% certolizumab vs. 43% placebo) at week 26 [50]. Thus, there is no recommendation to use certolizumab in the current guidelines for FCD [14,15,16,19].

In a recent meta-analysis of six RCTs, including two trials of infliximab, one trial of adalimumab, one trial of CDP571 and two trials of certolizumab, 34% (90/267) of patients treated with anti-TNF achieved fistula remission compared with 16% (26/165) of patients who received placebos. The pooled RR was 2.01 (95% CI, 1.36–2.97, *p* < 0.001). In the maintenance therapy, fistula remission was evaluated in two studies (one for infliximab and one for certolizumab). In total, 35% (43/124) of patients treated with anti-TNF maintained remission compared with 18% (23/129) of patients receiving placebos, with a pooled RR of 1.94 (95% CI, 1.25–3.02, *p* = 0.003) [26]. Of note, a relatively high rate of placebo response was reported in patients with fistulizing CD among all of the RCTs; 15.6% of patients who received placebos completed fistula closure [51].

Furthermore, biosimilars of infliximab and adalimumab were approved for all indications of the originator product in treating IBD. A large nationwide prospective study reported 209 patients with CD treated with infliximab biosimilar, with 39% of those having perianal FCD. The efficacy and safety of the biosimilars were comparable with treatment using the originator. The clinical remission and response rates were 48% and 65% at 1-year follow-up [52].

In conclusion, current evidence from RCTs and real-world studies support the use of infliximab as the first-line biologic for inducing and maintaining fistula healing based on RCTs with fistula-specific endpoints, as recommended in European and ACG guidelines [14,19]. Adalimumab can be used as an alternative first-line or second-line biologic in patients with previous infliximab failure based on their similar efficacy in terms of fistula response, although the level of evidence is lower [16,53,54].

### 2.2. What Is the Optimal Timing for Anti-TNF Treatment in Fistulizing CD?

The practice guideline recommends that immunosuppressive therapy should be started after adequate seton placement [19]. Given the concern for worsening perianal infection, real-world data demonstrated a significant delay in the induction of biologic therapy, with a median period of over 6 months between the diagnosis of perianal fistula and the induction of anti-TNF therapy [55].

The current data suggests that an early combination of seton and anti-TNF is safe and reasonably effective in treating perianal FCD. In a retrospective study, 76 patients were started on infliximab either late or early (250 days vs. 12 days) after seton insertion for perianal CD. The results regarding complete remission and fistula or abscess recurrence were not different between the early and delayed groups [56]. Recently, a cohort of 117 patients confirmed that the early duction of infliximab after surgery could achieve a fistula healing rate and re-intervention rate comparable to a delayed induction [57]. Thus, the early initiation of anti-TNF is preferred over delayed therapy to prevent consequent complications from fistulas.

In patients who achieve a fistula response thanks to medical therapy, maintenance therapy is recommended to maintain the long-term clinical response. The discontinuation of anti-TNF treatment is not recommended due to a high risk of relapse. Approximately half of the patients relapsed after anti-TNF was stopped, and one-third of patients with relapse required surgery [58,59,60].

### 2.3. What Is the Evidence Regarding New Biologics in Fistulizing CD?

#### 2.3.1. Ustekinumab

There has been no RCT assessing fistula response as a primary endpoint for ustekinumab (UST). A post hoc pool analysis of major clinical trials investigating the efficacy of UST (the UNITI-1, UNITI-2 and CERTIFI studies) included 150 patients, 10.8% to 15.5% of whom had active fistulas treated with UST. The rate of complete fistula healing was 24.7% (37/150) in patients on UST compared with 14.1% (10/71) for placebo-treated patients (*p* = 0.073) at 8 weeks, and no difference was observed in terms of the fistula response rate between the groups [35].

A national cohort study from France included 207 patients who had failed anti-TNF therapy, with 30% having active or inactive perianal CD treated with UST. The majority of patients had failed multiple biological treatments (99% had previously been exposed to at least one anti-TNF and 28% to VDZ). In the patients with active perianal FCD at UST initiation, approximately 40% were in remission from their perianal FCD at 6 months without the need for new medical or surgical intervention. Amongst patients with a history of perianal CD but inactive disease, 22% had a recurrence of perianal disease. The probability of recurrence-free survival was 86.2% and 75.1% at weeks 26 and 52, respectively [36].

A retrospective study assessed clinical and radiologic responses to UST in 167 patients with CD. In 45 patients (26.9%) with perianal fistulas, 31.1% achieved complete healing, as demonstrated by pelvic MRI or contrast-enhanced pelvic ultrasound [37]. In a prospective observational study of 28 patients who had active perianal fistulas, 35.7% had complete clinical resolution and 14.3% had a clinical response after 24 weeks [38].

In a meta-analysis of nine studies, with a total of 396 patients treated with UST, the pooled fistula response was 41.0%, 39.7% and 55.9% at weeks 8, 24 and 52, respectively. Regarding fistula remission, the pooled proportions were 17.1%, 17.7% and 16.7% at weeks 8, 24 and 52, respectively [39]. A more recent meta-analysis included 25 studies, and 44% (92/209) of patients with active perianal fistulas had a clinical response within 6 months of follow-up, with 53.9% (85/152) of patients with a 1 year of follow-up achieving a clinical response [40].

#### 2.3.2. Vedolizumab

Similar to UST, the efficacy of vedolizumab (VDZ) on fistula healing has been assessed only in terms of subgroup analysis. The efficacy of VDZ for FCD was first presented by Feagan et al., where a post-hoc analysis of 153 patients with FCD were treated with maintenance therapy with VDZ using data from the GEMINI 2 trial. This study showed that a higher proportion of patients receiving VDZ achieved fistula closure at week 52 compared to those who received the placebo (31% vs. 11%, absolute risk reduction 19.7%, 95% CI −8.9 to 46.2). Although differences did not reach statistical significance, the authors concluded that this data supports the efficacy of VDZ in treating FCD [41].

In a real-world nationwide multicenter study, 151 patients with perianal CD were treated with VDZ (102 with active fistulas, and 99% had prior anti-TNF treatment). In patients with active perianal disease, 22.5% achieved clinical success, portrayed as no draining fistula for 6 months. Among the patients with setons at VDZ initiation, 9 of 61 patients (15%) had a seton successfully removed. In patients with inactive perianal CD at the baseline, the perianal disease recurred in 30.6% of them, and 22.4% needed dedicated treatments during 22 weeks [42]. In a recent meta-analysis of four studies that included 198 patients with active perianal FCD, 87% had failed anti-TNF therapy. VDZ treatment led to the healing of perianal fistulas in 28% of the patients [43].

Moreover, a recent RCT (ENTERPRISE study) showed that an additional dose of VDZ at week 10 to the standard regimen does not appear superior to the standard intravenous dose for fistula healing, suggesting no benefit in terms of increased alternative dosing of VDZ for FCD [61].

#### 2.3.3. New Biological and Small Molecules Therapy Options for Fistulizing CD

The new therapeutic options have been actively explored and may have a role in the treatment of FCD in the future, with these including JAK inhibitors (filgotinib), IL-23 inhibitors (guselkumab) or IL-36 (spesolimab).

Filgotinib (FIL) is a once-daily, oral JAK-1 inhibitor. The efficacy of FIL for the treatment of perianal FCD was evaluated in a phase 2 RCT (DIVERGENCE 2 study) of 57 patients with perianal FCD, with 65% of the patients having failed to respond to anti-TNF. The patients treated with FIL 200 mg achieved a numerically higher rate of fistula response (FIL 47.1% vs. placebo 25.0%) and fistula remission (FIL 47.1% vs. placebo 16.7%) than the placebo group at week 24 [44]. Guselkumab has shown efficacy for luminal CD in a phase II trial, but efficacy for FCD treatment is warranted [62].

Thus, current evidence supports the use of ustekinumab and vedolizumab for fistulizing CD, including those patients who have failed/had an inadequate response to anti-TNF therapy. There is insufficient evidence to recommend optimal sequencing between ustekinumab to vedolizumab. Due to the overall weaker evidence in these cases (i.e., post hoc analysis of RCTs or real-world studies), the European guideline had less strong recommendations for using these new biologics in FCD [14,15]. Filgotinib has shown to be efficacious in a recent RCT phase 2 study with a primary endpoint of treating perianal FCD; however, further studies are warranted.

### 2.4. Do You Need to Use Combination Therapy for Optimizing Outcomes in Fistulizing CD?

Despite the widespread use of anti-TNF as the treatment of choice for CD, there is an unmet need in terms of treating FCD. Only one-third of patients treated with anti-TNF achieved short-term remission, and approximately 40% of patients who had an initial response maintained fistula healing remission in the long term [26,63]. Current studies have focused on treatment strategies to enhance the efficacy of anti-TNF therapy for fistula healing, including the use of combination therapy involving the use of anti-TNF with antibiotics, immunomodulators, therapeutic drug monitoring (TDM) and setons drainage [58].

#### 2.4.1. Combined Anti-TNF and Antibiotics

Three RCTs assessed the efficacy of antibiotics as adjuvant therapy to anti-TNF in perianal CD. In the ADAFI study, a randomized, placebo-controlled trial consisting of 76 CD patients with active fistulas, Dewint et al. showed that combining ciprofloxacin and ADA for 12 weeks is more effective than ADA alone in achieving fistula closure. At 12 weeks, the clinical response, defined as 50% fistula closure, was higher in the combination group compared to the group treated with ADA plus a placebo (71% vs. 47%, *p* = 0.047), with the same being true for completed fistula closure (65% vs. 33%, *p* = 0.009). However, after the discontinuation of ciprofloxacin, this effect was not maintained at 24 weeks [31].

Nonetheless, the two studies showed no significant difference, and the combination of ciprofloxacin and anti-TNF tended to be more effective than the use of anti-TNF alone. In a small randomized, placebo-controlled study in 24 patients, there was no significant reduction in the number of draining fistulas between the combination therapy and the placebo (8/11, 73% vs. 5/13, 39%, *p* = 0.12) [23]. Similarly, post hoc analysis from the CHARM study showed no significant difference in fistula healing when anti-TNF therapy was combined with antibiotics (27% vs. 33%) [30]. A recent meta-analysis suggested that the fistula response is significantly superior when antibiotics are combined with anti-TNF compared to anti-TNF alone (RR, 1.58; 95% CI, 1.09–2.28; *p* = 0.01) [26].

In summary, the addition of antibiotics to anti-TNF is more effective than anti-TNF alone in inducing a short-term fistula response but not in maintaining remission in the long term. Current practice guidelines recommend the initial use of antibiotics combined with anti-TNF in the treatment of active fistulizing CD [14,16,17,19]. Of note, for patients with perianal abscesses or complex fistulas, parallel seton or surgical drainage should be considered to control sepsis and fistula complications [19].

#### 2.4.2. Combined Anti-TNF and Immunomodulators

The efficacy of immunomodulators in fistula healing has not been evaluated as the primary endpoint in RCT. Therefore, data regarding the utility of thiopurine is derived from a sub-analysis of RCTs. A meta-analysis showed no statistical difference in fistula response between thiopurine monotherapy and placebos (30% vs. 16%, *p* = 0.2) [26]. Although thiopurines do not have a role as monotherapy in FCD, they can be used as an adjunct therapy to anti-TNF for synergistic effects, reducing immunogenicity and increasing levels of anti-TNF [64]. Nevertheless, the combination of immunomodulators with non-anti TNF biologics (VDZ or UST) is not more effective than biologic monotherapy for improving clinical outcomes in patients with CD [65,66].

A large retrospective cohort of 156 patients with perianal FCD were treated with IFX and of those, 56% received concomitant immunomodulators (thiopurine 91% or methotrexate 9%). At five years follow-up, 55% had complete closure. Multivariate analysis revealed that the combined therapy of IFX and immunomodulators in patients who were naïve to immunosuppressants was associated with a higher rate of fistula closure (HR 2.58, 1.16–5.6; *p* = 0.02) [24]. Furthermore, a later retrospective study also confirmed concomitant treatment with azathioprine increased fistula healing rates compared with IFX alone (50% vs. 36.9%, *p* < 0.001) [25]. Despite a retrospective study, this study featured a larger number of patients and had a long-term follow-up, supporting the adjunctive role of thiopurines in anti-TNF therapy in FCD.

In contrast, the results of a subgroup analysis of two RCTs comparing anti-TNF (IFX or ADA) and placebos showed no significant difference in fistula healing between anti-TNF monotherapy and concomitant therapy [22,30]. Similar findings were reported in observational cohorts [67,68]. Of note, in patients who had failed prior immunomodulator therapy, combined anti-TNF therapy has not been shown to be more effective than anti-TNF monotherapy [69].

Most guidelines suggest that a combination of thiopurine and anti-TNF may be effective and could be considered in the treatment of FCD in thiopurine naïve patients [16,19]. Of note, there is no recommendation supporting the use of thiopurine as a combination therapy in the European guideline [14].

#### 2.4.3. Combination of Dual Biologics Therapy

The combination of two biologics may be effective in treating patients with refractory CD [70,71,72]. In a meta-analysis of 279 patients who had previously failed biologics, 76% of whom had luminal CD, and who were treated with dual biologics, i.e., combined anti-TNF and VDZ (48%) or UST and VDZ (19%), approximately 60% of patients had clinical remission. However, this study did not assess patients with FCD [73]. A small retrospective cohort investigated 22 patients treated with dual biologics, and 33% of patients with perianal fistula had fistula healing after treatment [74]. These studies suggest a combination of two biologics may be an alternative option for treating refractory FCD. However, further studies are needed to solidify this strategy.

### 2.5. Are Biologics, Immunosuppressives and Combination Therapy Safe to Use in Fistulizing CD?

Patients with FCD have a high risk of recurrent perianal abscess or sepsis. Biological therapy and immunosuppressants are used for controlling active fistulas and inducing healing. Eventually, corticosteroids may be needed in some patients with active concomitant luminal disease. However, there is an increased concern that the use of biologics, immunosuppressive or combination therapy may increase the risk of serious infection, sepsis, hospitalization and need for urgent surgery [1,75,76].

In a meta-analysis of 15 observational studies, the risk of serious infection increased with the combination of anti-TNF and an immunosuppressive agent (in six cohorts; RR, 1.19; 95% CI, 1.03–1.37), especially in the case of anti-TNF and corticosteroids (in four cohorts; RR, 1.64; 95% CI, 1.33–2.03) or the use of all three drugs (in two cohorts; RR, 1.35; 95% CI, 1.04–1.77) compared to anti-TNF monotherapy [77]. A network meta-analysis of patients with active CD also showed no significant difference regarding the rate of adverse events and infections or serious/severe infections among biological/small molecule therapies (IFX, ADA, VDZ, UST, tofacitinib and CT-P13) [78].

In practice, only systemic corticosteroids should be avoided in patients with FCD or perianal abscess, given the higher risk of severe infections. Biologics and thiopurines are a safe combination therapy when combined with careful monitoring for perianal infection.

### 2.6. Is Therapeutic Drug Monitoring (TDM) Helpful in Optimize Treatment for Fistulizing CD?

Multiple studies have shown a correlation between higher anti-TNF levels and higher rates of perianal fistula response or closure [79,80,81,82,83]. In a post hoc analysis of the ACCENT-II trial, 282 patients with FCD received IFX for induction and 139 patients received maintenance therapy. Higher post-induction IFX concentrations were independently associated with remission at week 14 (OR: 2.32; 95% CI: 1.55–3.49) and at week 54 (OR: 2.05; 95% CI: 1.10–3.82). An IFX threshold of 15 μg/mL at week 6 and 7.2 μg/mL at week 14 stratified patients who achieved early fistula remission [45].

In a large cohort of 117 patients with perianal FCD, Yarur et al. reported that patients who achieved fistula healing had significantly higher trough IFX levels when compared to those who did not achieve healing (15.8 μg/mL vs. 4.4 μg/mL, *p* < 0.0001). In addition, the cuff off IFX level ≥ 10.1 μg/mL and > 20.3 μg/mL had a likelihood of achieving fistula healing 3- and 8-fold, respectively, when compared to those with IFX levels below 2.8 μg/mL [46].

Similar findings were noted for adalimumab levels. Patients with fistula closure had significantly higher drug levels than those without fistula closure (adalimumab:14.8 vs. 5.7 μg/mL, and IFX: 8.2 vs. 3.2 μg/mL). This study identified a cut off level > 7.1 μg/mL with IFX and >9.8 μg/mL with adalimumab for fistula closure [84].

In a more recent study of 193 perianal FCD patients treated with maintenance IFX or ADA, patients with MRI remission had higher anti-TNF levels compared with those with active disease (IFX 7.4 vs. 3.9 μg/mL; and ADA 9.8 vs. 6.2 μg/mL) [47].

Current evidence suggests that higher anti-TNF drug levels correlate with clinical and radiologic fistula remission. However, this causality is not proven. The optimal cut-off trough level varied amongst the studies. The target drug levels in fistulizing disease may be higher than conventional drug levels in luminal CD. The benefit of proactive TDM optimization is not proven in patients with FCD.

### 2.7. Is a Seton Required in the Long-Term or Short-Term as a Combination Therapy?

A seton is recommended as an adjunct to medical therapy for providing adequate fistula tract drainage to prevent recurrent abscess formation. In a systemic review including 305 patients with seton drainage, the rates of complete fistula closure after seton drainage varied from 13.6–100% and the fistula recurrence rate was up to 83.3% [85]. Multiple studies have shown the combination of a seton with IFX has a better fistula healing closure rate and lower recurrent rates of perianal fistulas and abscesses [56,86,87,88]. Thus, guidelines recommend using setons in combination with anti-TNF for FCD [19,89].

The optimal timing for seton removal is debatable. Seton drainage appears to promote fistula healing in combination with anti-TNF. However, prolonged drainage may interfere with the closure of the fistula. In a large retrospective study of patients treated with maintenance infliximab therapy, the cumulative rates of fistula recurrence were 12% and 36% at 1 and 5 years, respectively. The duration of seton drainage less than 34 weeks was associated with a higher fistula closure rate (HR 2.31), suggesting that a longer duration of seton placement does not prevent abscess recurrence [24]. One study reported that 14 patients treated with IFX and seton drainage successfully completed seton removal after five doses of infusions without early recurrence [90].

Recently, a randomized control study (PISA study) by Wasmann et al., the first of its kind, reported on 44 CD patients with a single internal opening perianal fistula. Patients were randomized into three treatment groups: chronic seton drainage for 1-year, anti-TNF therapy for 1 year and surgical closure after 2 months under a short course of anti-TNF. The seton treatment was associated with the highest re-intervention rate [10/15 seton vs. 6/15 anti-TNF vs. 3/14 surgical closure patients, *p* = 0.02]. There were no substantial differences in perianal disease activity and quality of life among the groups. This study showed an inferior outcome with respect to chronic seton treatment concerning re-interventions, suggesting that chronic seton treatment should not be recommended as the only treatment for perianal CD [91].

According to the result from the published literature, short-term seton placement should be considered as a bridging therapy and in combination with anti-TNF to control sepsis and promote fistula healing. However, setons should be removed after the induction of anti-TNF therapy, and their use should not exceed one year [17,92].

## 3. Medical Treatment for Non-Perianal Fistulizing CD

There is limited evidence to guide the management of non-perianal FCD due to a lack of established consensus guidelines. However, its management can be divided into immediate and maintenance treatment. The general principle of immediate treatment is similar in all types of non-perianal FCD: the controlling of infection and sepsis with antibiotics, fluid resuscitation, electrolyte rebalancing, nutritional support and prioritized surgical treatment if indicated [4].

In the acute phase of the disease, penetrating fistula and abscess formation may occur. Controlling sepsis with adequate antibiotics is needed in the initial management. Urgent surgical treatment is required in patients with free perforation or severe sepsis and no response to medical treatment [93]. In patients with the presence of intra-abdominal or retroperitoneal abscesses, radiologic guided percutaneous drainage (PCD) is preferred as a minimally invasive intervention to avoid surgical stoma formation [4,53,89]. With adequate drainage, half of patients indicated for surgery may avoid surgical operation for up to 2 months, allowing time to optimize nutritional support. However, 30% of patients fail PCD within 2 weeks and 25% have recurrent abscesses within 3 years. This suggests the benefit of PCD in selected patients, which could delay or prevent surgery in the short term. After adequate antibiotics and PCD, patients with uncontrolled or ongoing sepsis should undergo early surgical treatment [94,95,96].

Furthermore, the use of exclusive enteral nutrition (EEN) as adjunctive therapy for delaying or avoiding the need for surgery has the benefit of promoting fistula closure in patients with intestinal penetrating or stricturing or abscesses. In a prospective study of EEN in 41 patients with intestinal fistulas or intraabdominal abscesses, fistula closure after 12 weeks of EEN without biologic treatment was observed in 75% of patients with entero-cutaneous fistulas [97,98]. In addition, in a retrospective study of 55 patients with enterocutaneous fistulas, pre-operative EEN reduced the rate of postoperative intra-abdominal septic complications after bowel resections [99]. Thus, EEN should be considered in patients with malnutritional status, intestinal strictures or extensive intestinal inflammation.

In maintenance therapy, the specific treatment of non-perianal FCD depends on the anatomical location of fistulas and the connected organs. Biological therapy and/or an immunomodulator are recommended to control disease activity [89]. However, non-perianal fistulas are usually less responsive to medical therapies than perianal fistulas. Therefore, closed monitoring and early surgical management are recommended in patients with worsening symptoms or a lack of improvement after appropriate medical therapy.

Data on medical therapy for non-perianal fistulas remain lacking compared to for perianal fistulas. There has been no RCT investigating the effect of medical treatment specifically for non-perianal FCD. Most of the evidence comes from the subgroup analysis of previous RCTs and is limited by a small number of patients analyzed for this type of fistula. Most existing studies investigated the efficacy of anti-TNF and/or immunomodulators. Current guidelines favor anti-TNF. However, the recommendations are based on a low level of certainty concerning the evidence [14,15,19,100]. In this review, we discuss medical treatment for the different types of non-perianal FCD. Studies regarding medical therapy are summarized in Table 2.

### 3.1. Internal Fistula of GI Tract

Internal FCD of the GI tract includes entero-enteric, entero-colonic, gastro-enteric and gastro-colonic fistulas. The largest study assessing biological therapy in these types of FCD was a retrospective cohort that included 156 patients treated with anti-TNF therapy, 44% of whom had enteroenteric fistulas. The fistula healing rates according to MRI were 15%, 32% and 44% at 1, 3 and 5 years, respectively. However, 43% of patients required major abdominal surgery during the 3.5-year follow-up, and 20% of patients developed an intestinal abscess [101]. In another cohort of 93 patients treated with anti-TNFs, with 77% of patients having enteroenteric/colonic fistulas, the fistula closure rate and the surgery rate were 27% and 47% at 5 years from the induction of anti-TNF therapy, respectively [102]. Only case series data are available for non-biologic therapy. In a systematic review of seven studies, with 14 patients treated with thiopurine, 65% of patients had fistula healing [4].

### 3.2. Enterovesical Fistula

In a large observational cohort study of 97 patients with enterovesical (EV) fistulas, 33 patients (35%) were treated with anti-TNF and 45% of them achieved remission without needing surgery over a follow-up period of 35 months. The use of anti-TNF therapy was associated with an increased remission rate without the need for surgery (HR 0.23, 95% CI 0.12–0.44). However, 80% of patients required surgery over a median follow-up of 8 years [103]. In a systematic review, the anti-TNF treatment of 14 patients with EV fistulas resulted in 57% of them having a complete response and 35% having a partial response [104]. In addition, antibiotics have an important role to play in the symptomatic control and treatment of urosepsis or associated abscesses. Thiopurine, used with or without long-term antibiotics in 99 entero-urinary fistulas, resulted in an overall response of 39.4% [4].

### 3.3. Rectovaginal Fistula

Rectovaginal (RV) fistulas may cause UTIs, abscess formation and fecal incontinence. The use of antibiotics can improve short-term symptoms, especially in patients with recurrent UTIs [108]. In terms of biological therapy, the efficacy of anti-TNFs on RV fistulas was demonstrated in a systemic review reporting on 78 patients with RV fistulas who were treated with anti-TNF alone or combined with antibiotics or immunosuppressants. The overall response rate was 63%, and 37% of patients had no response [104]. In 2018, a large national-wide retrospective cohort reported on 204 patients with RV fistulas who received anti-TNF (79% infliximab, 20% adalimumab and 1% certolizumab), where 37% had complete fistula closure and 22% had a partial response at 1-year follow-up [105]. One case report of combined dual biologic therapy with VDZ and UST showed a successful fistula closure in a patient with a refractory RV fistula [109]. Thiopurines and 6-MP were reported to have efficacy in a small group of patients, with an overall response rate of 50% in 12 reported cases [110,111]. Cyclosporin was shown to be effective in the induction phase for RV fistulas, with an 89% improvement in nine patients. However, the relapse rates were reported to be 38% of responders during the maintenance phase [112]. The efficacy of tacrolimus has also been demonstrated in two out of three patients with refractory RV fistulas who had failed other therapies, including infliximab [113].

### 3.4. Enterocutaneous Fistula

Enterocutaneous (EC) fistulae can occur primary or secondary to surgery and can be complicated by malnutrition, coexisting intraabdominal infection, abscesses or a high output of small bowel content, with the majority of affected patients requiring intestinal surgery [53]. Anti-TNFs seem to have limited efficacy only after sepsis has been resolved and when intestinal stenosis is excluded. In a retrospective cohort of 48 CD patients with EC fistulas, 33% of patients had complex fistulas with multiple tracts and 23% had high output. Regarding the fistulas treated with anti-TNF (infliximab; 78%), 33% of patients achieved completed fistula closure. However, half of the patients failed to avoid surgery after 2 years. Stenosis, complex fistulas and concomitant steroids were significant predictors for anti-TNF failure [106]. A similar rate of fistula closure was reported in a cohort of eight patients treated with IFX; in total, 38% of patients experienced the complete cessation of EC fistula drainage [107].

In conclusion, the data on medical treatment for non-perianal FCD are scarce. Despite limited studies, anti-TNF was noted to have the most evidence in for treating non-perianal FCD in selected patients [53]. However, there is a high risk of non-response, and the likelihood of the need for surgery is high. Therefore, surgery is still the mainstay of treatment in the long term. Medical therapy can reduce the pre-operative disease severity and postoperative disease recurrence [114]. Nonetheless, there is insufficient data to recommend the use of immunomodulators alone or non-anti-TNF agents in non-perianal FCD [4,17,19].

## 4. Surgical Management of Fistulizing CD

The surgical management of FCD requires a multidisciplinary approach, including controlling sepsis, fistula closure and inflammation as discussed. Patients with active perianal fistulizing disease commonly present a perianal abscess. Along with medical therapy, the surgical drainage of these abscesses is vital to prevent sepsis. Symptomatic patients, e.g., those with recurrent UTIs or obstructive symptoms, require surgical management. Furthermore, surgery should be considered at an early stage in asymptomatic FCD patients not responding to medical therapy, as chronic inflammation can involve otherwise healthy small and large bowels in the long run [115]. Table 3 provides a summary of selected studies of surgical management and/or in combination with medical therapy in patients with perianal fistulizing CD.

### 4.1. Seton Drainage

To maintain the patency of fistula tracts, preventing the premature closure of the external orifice and thus limiting recurrent abscess formation, seton placement has been shown to be effective [121]. There are two techniques when it comes to seton placement, with the preferred technique being the loose, non-cutting seton technique, which avoids the risk of incontinence and perianal pain [122,123].

A meta-analysis has shown short-term successful fistula healing in the use of setons alone, ranging from 14–81%, with long-term fistula recurrence reported in 47% [124]. Long-term setons can be considered for symptom control in patients with recurrent perianal abscesses to avoid proctectomy [125]. Nevertheless, a recent RCT suggests that chronic setons alone should not be used for treating perianal fistulas. [91]. The guidelines recommend seton placement in combination with anti-TNF therapy for improving the healing of perianal FCD [17,19,53].

### 4.2. Fistulotomy and Ligation of the Intersphincteric Fistula Tract (LIFT)

For patients with low intersphincteric and low trans-sphincteric fistulas, a fistulotomy or lay-open are safe and effective methods of choice, with an 81–100% healing rate and low post surgical fistula recurrence. A fistulotomy is usually performed after the failure of medical treatment and no active proctitis [126,127]. However, this procedure is associated with fecal incontinence, reported in 60% of patients. If the fistula is located more anteriorly, fistulotomy should be avoided due to the risk of incontinence [128].

A study by Van Onkelen et al. demonstrated that trans-sphincteric fistulas can be treated by the ligation of the intersphincteric fistula tract, a procedure called “LIFT”, if they have matured into fibrotic tubes with granulation tissue, as that enables ligation and transection. Furthermore, “LIFT” preserves the sphincter and reduces the risk of incontinence, with comparable rates of healing to other methods [129,130].

### 4.3. Advancement Flap, Fistula Plug and Fibrin Glue

Management of high fistulas can be categorized into three different categories: the mucosal advancement flap and the filling of the perianal fistula tract with bioprosthetic plugs or glue. The mucosal advancement flap involves mobilizing rectal tissue to cover the internal opening of the fistula tract, without affecting the sphincter complex. A systematic review showed that the average success rate of the mucosal advancement flap after a mean follow-up of 29 months was 64% (range 33–92%), with reoperation needed in 50% of patients [131]. Furthermore, this procedure can be used for rectovaginal fistulas, although their recurrence rate is higher.

Bioprosthetic plugs made of collagen or porcine intestinal submucosa have been shown to be effective in closing fistulas [132,133,134]. A systematic review of 20 studies showed wide variations in fistula closure, ranging from 15 to 86%, with pooled closure rates of 55% in patients with CD [124,135]. Nevertheless, a randomized open-label trial showed that fibrin plugs were not more effective than seton removal alone in achieving fistula closure.

Fibrin glue, which is made up of fibrinogen and thrombin, when injected into a fistula tract, forms a clot in the lumen to help to seal the tract. A randomized open-label of 77 patients with perianal FCD reported a higher rate of fistula healing in patients who received fibrin glue compared to the observation group (36% vs. 16%, OR 3.2) [136]. However, the benefit was more significant only in patients with simple fistulas, and real-world observational studies reported a large variation in fistula healing rates, ranging from 40–67% [124,137,138,139,140]. Another recent RCT compared seton removal plus surgical closure (79% had fibrin glue) to seton removal alone in 64 patients with perianal FCD treated with adalimumab. The fistula closure rate was not significant between the groups (surgical closure plus seton 56% vs. seton alone 65%) [119]. Of note, fibrin glue is not effective in patients with complex fistulas [136], even when combined with other modalities such as antibiotics [141].

### 4.4. Fecal Diversion and Proctectomy

Fecal diversion and a diverting stoma can be considered for patients with severe sepsis or those who have not responded to drainage or seton placement. It can also be used as a temporary method to improve the overall condition of patients and their quality of life up to the point that they receive medical therapy or proctectomy [142,143]. In a meta-analysis, 64% of patients had an early clinical response after fecal diversion for refractory perianal CD. Restoration of bowel continuity was successful in only 17%, and 26.5% of those who had restoration required re-diversion because of severe relapse. Overall, 42% of patients required a proctectomy after the failure of temporary fecal diversion [142].

Proctectomy with a permanent stoma is used in those patients that have failed prior therapies and hence is only used as a final alternative for severe refractory perianal FCD. Studies have shown that it is required in 10–20% of patients with refractory disease [144,145]. Complications, such as poor wound healing and perineal sinus formation, may occur in up to 25–50% of patients [146,147]. To prevent this, the transposition of the gracilis is an option. This could also be used before a proctectomy for patients with other types of Crohn’s-related or complicated fistulas for whom other treatments have failed [148].

### 4.5. Mesenchymal Stem Cell Therapy

In the last decade, stem cell-based therapy has emerged as an attractive new therapeutic option for patients with FCD. Adipose-derived stem cells of mesenchymal origin are activated in an inflammatory environment (e.g., fistula). An early RCT demonstrated their success in healing the fistulas, with 71% of patients receiving stem cell therapy, but the long-term follow-up showed a recurrence rate of 17.6% [149,150].

Most recently, the ADMIRE-CD Phase 3 RCT demonstrated the efficacy of stem cell therapy, namely darvadstrocel administration, used once, locally in patients who did not respond to conventional and/or biological treatments. Clinical and MRI remission was achieved and was significantly greater in patients treated with darvadstrocel 51.5% (53/103) compared to the placebo group 35.6% (36/101) at 24 weeks follow-up [116]. At 2 years follow-up, 56% of patients had sustained remission [117].

A recent real-world retrospective cohort study (INSPECT study) reported that 54% (23/43) of patients in the ADMIRE-CD trial remained in remission at 3 years follow-up, with a favorable safety profile, i.e., treatment-related tumorigenicity [118]. Alofisel^®^/darvadstrocel is approved for use for this purpose in the European Union [151].

The efficacy of autologous adipose tissue-derived stem cell (ASC) transplantation was compared to anti-TNF after seton placement in the retrospective study of 69 patients with perianal FCD. The closure rate was faster, and the time to closure was significantly shorter in patients who underwent auto-ASC transplantation when compared to anti-TNF treatment (14 vs. 37 months) [152].

A recent meta-analysis of 29 studies demonstrated that stem cells had a higher rate of fistula healing compared to placebos (61.75% vs. 40.46%, OR; 2.21, 95% CI 1.19–4.11, *p* < 0.05), suggesting that stem cell therapy is a promising method in the treatment of FCD, especially the use of adipose-derived mesenchymal stem cells [153].

In addition, multiple advanced surgical methods, such as endoscopic fistulotomy, intra-lesional anti-TNF injection, fistula-tract laser closure (FiLaCTM), video-assisted anal fistula treatment (VAAFT) and hyperbaric oxygen therapy, may be effective alternative treatments for complex refractory fistulas. However, there have only been a small number of patients included in the reported studies on these techniques. Larger studies and longer follow-up results are warranted before suggesting the use of these methods [154,155].

In conclusion, the optimal surgical approach for fistulizing CD in clinical practice can differ among individuals, with there being no consensus regarding the best surgical approach. Therefore, the type of surgical procedure should be tailored to the anatomical location of the fistulas and the surgeon’s expertise. Seton placement should be combined with anti-TNF therapy to induce fistula healing. Fistulotomy or LIFT may be considered in patients with symptomatic low-simple fistulas without active proctitis or abscess. For complex fistulas (those that are high up, involving muscle, associated with strictures or abscesses and multiple opening fistulas), LIFT, an advancement flap, fibrin glue or fistula plugs (limited efficacy) can be considered. Recently, mesenchymal stem cell therapy has been approved, with promising efficacy in patients with medical refractory fistulizing CD. Lastly, proctectomy or diversion may be required in patients with severe uncontrolled perianal fistulas [144].

## 5. Optimizing Strategies for Medical and Surgical Treatment of Fistulizing CD

**Figure 1:**
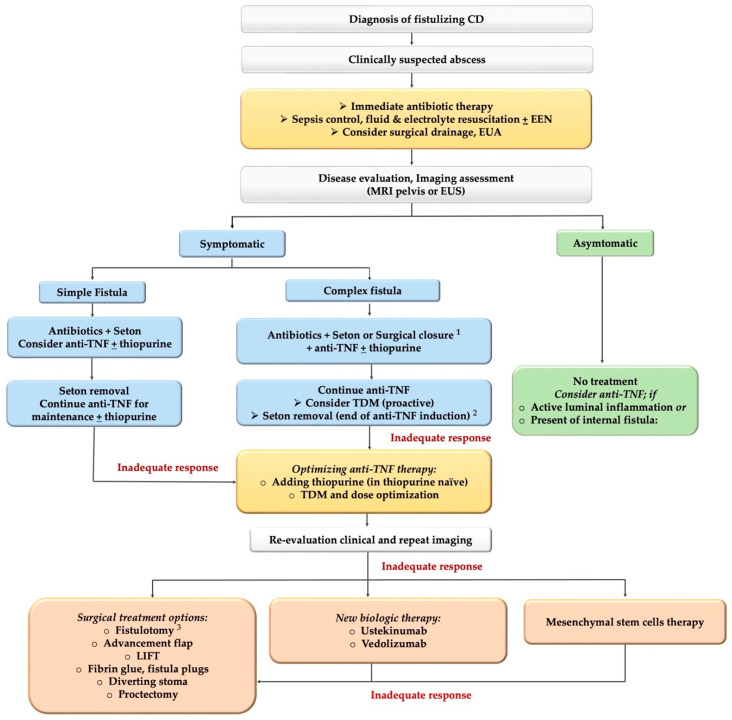
The proposed treatment algorithm for the management of fistulizing Crohn’s disease. Note: 1. Surgical closure for single opening fistula may used instead of seton. 2. Seton removal should be considered after induction of anti-TNF with adequate response (no abscess or sepsis). 3. Fistulotomy should be considered only in simple, low fistula without active protitis/perianal abscess. Abbreviations; EUA: examination under anesthesia, EUS: endoscopic ultrasonography; EEN: exclusive enteral nutrition, TDM: therapeutic drug monitoring, LIFT: ligation of the intersphincteric fistula tract.

The combination of surgical treatments (e.g., seton, fistulotomy, advancement flap, fibrin glue) with medical therapy (anti-TNF with or without immunomodulators) has shown a better overall fistula healing response compared with surgery or medical therapy alone. In a systematic review including 1139 patients, complete fistula remission as a result of combination therapy was higher than for single therapy (52% vs. 43%) [120]. Therefore, a multidisciplinary approach involving gastroenterologists, surgeons, radiologists and nutritionists is required to optimize the treatment outcome using this strategy. The proposed treatment algorithm for the management of FCD is shown in Figure 1.

It is necessary to rule out a perianal abscess in patients with fistulas and perianal pain or mass with signs of sepsis. If this is clinically suspected or detected, immediate antibiotics therapy and urgent surgical consultation for examination under anesthesia (EUA) combined with adequate drainage should be performed to control the sepsis and prevent the destruction from the undrained abscess. Small perianal abscesses may not require surgical drainage [16,17,19,89].

The patients with asymptomatic fistulas do not require treatment or may be considered for biologic therapy if there is active luminal inflammation, given that FCD indicates a severe and aggressive disease phenotype [53,156].

Symptomatic simple perianal fistulas can be managed using a combination of antibiotics and anti-TNF to induce fistula healing. In non-responded patients, a seton or surgical closure by fistulotomy should be considered after ruling out active proctitis. However, fistulotomy is commonly associated with significant postoperative incontinence. The guidelines suggest this procedure may be performed in only low, simple or superficial fistulas [17,19,53].

For complex perianal fistulas, a combination of medical therapies, including anti-TNF, antibiotics and surgical intervention (seton placement or surgical closure), should be considered for inducing and maintaining fistula healing [6,16,17,19,53]. Loose setons can also be used as a bridging therapy between medical and definitive surgical treatment. Nonetheless, setons should be removed to allow for fistula closure after inducing infliximab therapy. Current evidence has shown no additional benefits regarding chronic seton treatment compared to anti-TNF, and chronic seton treatment is associated with a higher re-intervention rate [18,91].

In case of non-response, immunomodulators (azathioprine or 6-MP) can be added to enhance the efficacy of anti-TNF therapy in thiopurine-naïve patients. Furthermore, the TDM of anti-TNF therapy is suggested for dose optimization. In patients with anti-TNF failure, definitive surgical treatment should be discussed. The local injection of mesenchymal stem cells therapy can be performed in an experienced center with available resources. Switching to other anti-TNF (ADA) or new biological therapies (UST or VDZ) in the patients who do not respond to IFX is an alternative option in selected patients to avoid surgical treatment. The commencing of dual biologics therapy is reasonable for multiple biologics failure. Fecal diversion with a stoma or proctectomy may be considered to increase fistula healing and prevent further structural damage in severe refractory FCD [14,15,18,19,157].

## 6. Conclusions

Concerning the current management of FCD, evidence based on RCTs is insufficient when it comes to contributing to the best strategies for this phenotype of CD. Nonetheless, multiple recent studies beyond non-RCTs provide more solid evidence for guiding physicians in clinical practice. This review highlights the combination of medical and surgical therapy as the most effective strategy in treating patients with FCD. Based on a level I evidence from two RCTs, anti-TNF therapy remains the cornerstone and is recommended as the first-line biological treatment for FCD.

To optimize the efficacy of anti-TNF therapy, concomitant antibiotics and/or combined with an immunomodulator, seton placement and TDM with dose optimization are efficacious in improving fistula healing, meaning that medical and surgical approaches should be combined. In addition, the early initiation of anti-TNF is preferred over delaying therapy to minimize fistula complications. Chronic setons should not be used as a single therapy and they should be removed after anti-TNF induction to allow for fistula closure.

UST and VDZ were shown to be efficacious as second-line biologics in patients who had an inadequate response or failed anti-TNF. However, it is important to note that the results from post hoc analyses might not represent the true efficacy of biological therapy, and the risk of bias given randomization was not stratified by the presence or absence of fistulas. In the RCTs, primary indication of biologic therapy was not to treat fistula healing but for active luminal CD. More recently, an emerging novel treatment in mesenchymal stem cell therapy has been shown to be promising and was approved for the treatment of medically refractory patients.

## Figures and Tables

**Table 1:** Summary of selected studies of medical therapy in fistulizing Crohn’s disease.

Study, Year	Study Design	No.	Fistula Type (%)	Intervention and/or Comparator	Study Endpoint (Duration)	Main Result
Infliximab (IFX)						
Present et al., 1999 [21]	RCT	94	Perianal (90%) Abdominal (10%)	IFX (56) vs. PBO (29)	Fistula response: 50% closure, fistula remission: 100% closure (18 months)	Fistula response rate: IFX 62% vs. PBO 26% (*p* = 0.002) fistula remission rate: IFX 46% vs. PBO 13% (*p* = 0.001)
Sands 2004 (ACCENT II) [22]	RCT	282	Perianal (87%), abdominal (11%), rectovaginal (8%)	IFX (96) vs. PBO (99)	Time to the loss of response in maintenance phase (14 and 54 weeks)	IFX > 40 weeks vs. PBO 14 weeks (*p* < 0.001), complete response rate at 54 weeks: (IFX 36% vs. PBO 19%, *p* = 0.009).
West et al., 2004 [23]	RCT	24	Perianal (100%)	IFX + CIP (11) vs. IFX + PBO (13)	Response: 50% reduction in the number of draining fistulas (18 weeks)	The higher response rate in IFX + CIP; 73% vs. IFX + PBO; 39%, *p* = 0.12
Bouguen et al., 2013 [24]	Retrospective	156	Perianal (100%)	IFX + AZA (90) vs. IFX alone (66)	Fistula closure (5 years)	Combined therapy was associated with an increased rate of fistula closure (HR 2.58, 1.16–5.6; *p* = 0.02)
Zhu et al., 2021 [25]	Retrospective	178	Perianal (100%)	IFX (178)	Clinical and radiological (MRI) (135 weeks)	Clinical remission and response: 55.1% and 26.4%, radiological remission and response: 38.2% and 34.3%, respectively
Lee at al., 2018 [26]	Meta-analysis	432	Perianal and non-perianal fistulas	Six studies Anti-TNFs (267) vs. PBO (165)	Fistula remission and response	Induction remission rates; anti-TNFs 34% (90/267) vs. PBO 16% (26/165). Pooled RR: 2.01 (95% CI, 1.36–2.97, *p* < 0.001). In the maintenance therapy, fistula remission: anti-TNFs 35% (43/124) vs. PBO 18% (23/129), pooled RR: 1.94 (95% CI, 1.25–3.02, *p* = 0.003)
Adalimumab (ADA)						
Hanauer et al., 2006 (Classic I) [27]	RCT	32	Perianal, enterocutaneous (NS)	ADA (26) vs. PBO (6)	Response: 50% closure, remission: 100% closure (4 weeks)	No significant difference in fistula healing rates ADA vs. PBO
Sandborn et al., 2007 (GAIN) [28]	RCT	45	Perianal, abdominal (NS)	ADA (20) vs. PBO (25)	Response: 50% closure, remission 100% closure (4 weeks)	No difference in fistula response: ADA 15% vs. PBO 20% and remission: ADA 5% vs. PBO 8%.
Colombel J.F. et al., 2009 (CHARM) [29,30]	RCT and post hoc	117	Perianal (97%), abdominal (3%)	ADA (70) vs. PBO (47)	Fistula healing (100% closure) (2 years)	At 26 weeks: ADA 30% vs. PBO 13%, *p* = 0.043, and at 52 weeks: ADA 33% vs. PBO 13%, *p* = 0.016, and 90% of patients who had fistula healing maintained healing until 2 years
Dewint et al., 2014 (ADAFI) [31]	RCT	76	Perianal (100%)	ADA + CIP (36) vs. ADA (34)	Fistula response: 50% closer, remission: 100% closure (12 and 24 weeks)	Fistula response at 12 weeks: ADA + CIP 71% and ADA + PBO 47%, *p* = 0.047and remission: ADA + CIP 65% and ADA + PBO 33%, *p* = 0.009. The response was not maintained at 24 weeks.
Lichtiger et al., 2010 (CHOICE) [32]	phase IIIb single-arm	88	Perianal (93%), enterocutaneous (13%)	ADA (88)	Fistula healing: 100% closure (36 weeks)	Fistula healing rate: 39% during a follow-up visit (4–36 weeks)
Castaňo-Milla et el., 2015 [33]	Retrospective	46	Perianal (100%)	ADA (46)	Fistula healing and response (6 and 12 months)	Fistula responses: 72% and 49% of patients at 6 and 12 months, respectively
Ji and Takano et al., 2017 [34]	Retrospective	47	Perianal (100%)	ADA (16) vs. IFX (31)	Recurrence-free and disease progression (2 years)	No significant difference between IFX 83.9% vs. ADA 62.5%, *p* = 0.09
Ustekinumab (UST)						
Sand 2017 (UNITI-1, UNITI-2, CERTIFI) [35]	Posthoc analysis	238	Perianal (100%)	UST (161) vs. PBO (77)	Fistula healing: 100% closure, response: 50% closure (8 weeks)	Fistula healing: UST 24.7% vs. 14.1% PBO (*p* = 0.073); no significant fistula response between the groups.
Chapuis-Biron et al., 2020 (LioLAP) [36]	Retrospective	207	Perianal (100%)	UST 207	Success rate: no need for surgical or additional treatment (52 weeks)	In patients with active fistula, success rate: 38.5% (57/148), successful seton removal: 33% (29/88), and recurrence-free survival: 75.1%.
Ma et al., 2017 [37]	Retrospective	45	Perianal (100%)	UST (45)	Completed healing on imaging (12 months)	31.1% of patients achieved complete radiologic healing (MRI or contrast-enhanced pelvic ultrasound)
Biemans et al., 2020 [38]	Prospective observational	28	Perianal (100%)	UST (28)	Fistula response and remission (24 weeks)	Complete clinical remission: 35.7% and clinical response: 14.3%
Attauabi et al., 2020 [39]	Meta-analysis	396	Perianal (100%)	Nine studies UST (396)	Fistula response and remission (52 weeks)	The pooled fistula response: 41.0%, 39.7%, and 55.9% at weeks 8, 24, and 52, respectively. Pooled fistula remission: 17.1%, 17.7%, and 16.7% at week 8, 24, and 52, respectively.
Brewer et al., 2021 [40]	Meta-analysis	209	Perianal (100%)	25 studies UST (209)	Fistula response (6 and 12 months)	Clinical response: 44% and 53.9% at 6 and 12 months, respectively
Vedolizumab (VDZ)						
Feagan et al., 2018 (GEMINI 2) [41]	Post hoc analysis	57	Perianal (79%), NS (21%)	VDZ (39) vs. PBO (18)	Fistula closure; no drainage (14 and 52 weeks)	Fistula closure at week 14: VDZ 28% vs.. PBO 11% (ARR: 17.1%; 95% CI, −11.4 to 43.9). At week 52: VDZ 33% vs. PBO 11% (ARR: 19.7%; 95% CI, −8.9 to 46.2)
Chapuis-Biron et al., 2020 [42]	Retrospective	151	Perianal (100%)	VDZ (151)	Clinical remission, seton removal and recurrence rate (6 months)	In patients with active fistula, clinical remission: 22.5%, successful seton removal: 9/61(15%), and in patients with inactive fistula, the perianal disease recurrence: 30.6%
Ayoub et al., 2022 [43]	Meta-analysis	198	Perianal (100%)	Four studies VDZ (198)	Complete and partial fistula healing	The pooled complete healing rate: 27.6% (95% CI, 18.9–37.3%), pooled partial healing rate: 34.9% (95% CI, 23.2–47.7%)
Filgotinib (FIL)						
Reinish et al., 2022 (DIVERGENCE2) [44]	RCT	57	Perianal (100%)	Filgotinib (42) vs. PBO (15)	Combined clinical and MRI response/remission (24 weeks)	Fistula response was numerically higher in the FIL 200 mg group (47.1%; CI: 26.0–68.9) vs. PBO group (25.0%; CI: 7.2–52.7). Fistula remission (FIL 200 mg (47.1%; CI: 26.0–68.9) vs. PBO (16.7%; CI: 3.0–43.8))
Therapeutic drug monitoring (TDM)						
Papamichael et al., 2021 [45]	Post hoc of the ACCENT-II	282	Perianal (87%) Abdominal (11%), rectovaginal (8%)	IFX level high vs. low	Fistula remission (54 weeks)	The higher post-induction IFX levels were associated with remission (OR: 2.05; 95% CI: 1.10–3.82). IFX level of 15 mg/mL at week 6 and 7.2 mg/mL at week 14 stratified early fistula remission
Yarur et al., 2017 [46]	Observational	117	Perianal (100%)	TDM level high vs. low	Fistula healing (29 weeks)	IFX levels: patients with healing: 15.8 ug/mL vs. no healing: 4.4 ug/mL, *p* < 0.001. The cut-off > 10.1 mcg/mL and >20.3 mcg/mL predict fistula healing 3- and 8-fold, respectively.
Gregorio et al., 2021 [47]	Retrospective	193	Perianal (100%)	TDM level in active vs. remission	Radiologic response on MRI (2.1 years ADA, 2.5 years IFX)	Anti-TNF levels in patients with MRI remission vs. active disease: IFX; 7.4 vs. 3.9 mg/mL; and ADA; 9.8 vs. 6.2 mg/mL.

Abbreviations: RCT: randomized controlled trial, TNF: tumor necrosis factor, IFX: infliximab, ADA: adalimumab, VDZ: vedolizumab, UST: ustekinumab, FIL: filgotinib, CIP: ciprofloxacin, AZA: azathioprine, PBO: placebo, TDM: therapeutic drug monitoring, ARR: absolute risk reduction, HR: hazard ratio, OR: odds ratio, NS: non-specified.

**Table 2:** Summary of selected studies of medical therapy in non-perianal FCD.

Study, Year	Study Design	No.	Treatments	Study Endpoints (Duration)	Main Results
1. Internal fistula of GI tract					
Bouguen et al., 2020 [101]	Retrospective	156	Anti-TNF, IFX (75%), ADA (25%)	Fistula healing and need for surgery (3.5 years)	The fistula healing rates on MRI were 15%, 32% and 44% at 1, 3, and 5 years, respectively. In total, 43.6% of patients required surgery in a period of 3.5 years
Kobayashi et al., 2017 [102]	Retrospective	93	Anti-TNF, IFX (74%), ADA (26%)	Need for surgery and fistula closure (5 years)	Surgery rate was 47.2% and fistula closure rate was 27.0% at 5 years. Only single fistulas were associated with successful fistula closure.
2. Enterovesical fistula					
Taxonera et al., 2016 [103]	Retrospective	97	Anti-TNF (35%)	Fistula remission by clinical and imaging (35 months)	In total, 45% of patients achieved remission without needing surgery (HR 0.23, 95% CI 0.12–0.44).
Kaimakliotis et al., 2016 [104]	Systematic review (five studies)	14	Anti-TNFs	Fistula closure	In total, 7.1% of patients had a complete response, 35.7% partial response and 7.1% no response.
3. Rectovaginal fistula					
Kaimakliotis et al., 2016 [104]	Systematic review (nine studies)	78	Anti-TNFs	Fistula closure and response (1 year)	A total of 41.0% of patients had complete response, 21.8% partial response and 37.2% no response.
Le Baut et al., 2018 [105]	Retrospective	204	IFX (79%), ADA (20%), certolizumab (1%)	Fistula closure and response (1 year)	A total of 37% of patients had complete fistula closure, 22% partial response and 41% no response. Only complementary surgery was associated with better response (RR 2.02, 95% CI: 1.25–3.26).
4. Enterocutaneous fistula					
Amiot et al., 2014 [106]	Retrospective	48	Anti-TNFs	Fistula closure (3 years)	In total, 33% of patients had complete closure, of whom 50% relapsed and 54% needed surgery
Parsi et al., 2004 [107]	Retrospective	14	IFX	Fistula closure (9 months)	A total of 38% of patients had complete cessation of EC fistula drainage

Abbreviations: TNF: tumor necrosis factor, IFX: infliximab, ADA;:adalimumab, RR: risk reduction, HR: hazard ratio.

**Table 3:** Summary of selected studies of surgical treatment alone or combined with medical therapy in perianal fistulizing Crohn’s disease.

Study, Year	Design	No.	Intervention and Comparator	Study Endpoint	Result
Wasmann et al., 2020 (PISA) [91]	RCT	44	IFX (15) vs. seton (15) vs. surgical closure (14)	Fistula related re-intervention (1.5 years) and disease activity	Seton treatment was associated with the highest re-intervention rate (10/15, vs. 6/15); anti-TNF and 3/14 surgical closure patients, *p* = 0.02. No differences in perianal disease activity and QoL between the three groups
Panés et al., 2016, 2022 (ADMIRE-CD and INSPECT) [116,117,118]	RCT and post hoc analysis	212	Darvadstrocel (107) vs. control (105)	Combined clinical and MRI remission (24, 52, 104 and 156 week)	RCT: at week 24, combined remission; darvadstrocel 50% vs. control 34%, *p* = 0.024 Post-hoc: at weeks 52, 104 and 156. Clinical remission 67.4%, 53.5% and 53.5% of 43 darvadstrocel-treated patients, compared with 52.2%, 43.5% and 45.7% of 46 in the control group, respectively.
Abramowitz et al., 2021 [119]	RCT	64	Surgical closure (33, 79% had glue) vs. control (31)	Fistula closure: no seton and no draining fistula (12 months)	Fistular closure: surgical closure 56% vs. control group 65%, *p* = 0.479. In the surgical closure group, fistula closure: 52% in complex and 71% in simple fistula.
Yassin et al., 2014 [120]	Meta-analysis (24 studies)	1139	Combination of medical with surgery (679) vs. medical/surgical alone (460)	Fistula healing	Complete remission rates: single therapy 43% vs. combination 52%.

Abbreviations; RCT: randomized controlled trial, TNF: tumor necrosis factor, IFX: infliximab.

## Data Availability

Not applicable.
